# Relationship between the Plasma Proteome and Changes in Inflammatory Markers after Bariatric Surgery

**DOI:** 10.3390/cells10102798

**Published:** 2021-10-19

**Authors:** Helene A. Fachim, Zohaib Iqbal, J. Martin Gibson, Ivona Baricevic-Jones, Amy E. Campbell, Bethany Geary, Akheel A. Syed, Antony Whetton, Handrean Soran, Rachelle P. Donn, Adrian H. Heald

**Affiliations:** 1 The School of Medicine and Manchester Academic Health Sciences Centre, Manchester University, Manchester M13 9PL, UK; zohaib@doctors.org.uk (Z.I.); martin.gibson@manchester.ac.uk (J.M.G.); ivona.baricevic-jones@manchester.ac.uk (I.B.-J.); amy.campbell@manchester.ac.uk (A.E.C.); bethany.geary@manchester.ac.uk (B.G.); akheel.syed@manchester.ac.uk (A.A.S.); tony.whetton@manchester.ac.uk (A.W.); handrean.soran@mft.nhs.uk (H.S.); Rachelle.donn@manchester.ac.uk (R.P.D.); 2 Salford Royal Foundation Trust, Department of Endocrinology, Diabetes and Metabolism, Salford M6 8HD, UK; 3 Stoller Biomarker Discovery Centre, Division of Cancer Sciences, School of Medical Sciences, Faculty of Biology, Medicine and Health, University of Manchester, Manchester M13 9PL, UK; 4 Manchester National Institute for Health Research Biomedical Research Centre, Manchester M13 9WL, UK

**Keywords:** bariatric surgery, SWATH-MS, proteomics, diabetes remission, inflammatory markers

## Abstract

Severe obesity is a disease associated with multiple adverse effects on health. Metabolic bariatric surgery (MBS) can have significant effects on multiple body systems and was shown to improve inflammatory markers in previous short-term follow-up studies. We evaluated associations between changes in inflammatory markers (CRP, IL6 and TNFα) and circulating proteins after MBS. Methods: Sequential window acquisition of all theoretical mass spectra (SWATH-MS) proteomics was performed on plasma samples taken at baseline (pre-surgery) and 6 and 12 months after MBS, and concurrent analyses of inflammatory/metabolic parameters were carried out. The change in absolute abundances of those proteins, showing significant change at both 6 and 12 months, was tested for correlation with the absolute and percentage (%) change in inflammatory markers. Results: We found the following results: at 6 months, there was a correlation between %change in IL-6 and fold change in *HSPA4* (rho = −0.659; *p* = 0.038) and in *SERPINF1* (rho = 0.714, *p* = 0.020); at 12 months, there was a positive correlation between %change in IL-6 and fold change in the following proteins—*LGALS3BP* (rho = 0.700, *p* = 0.036), *HSP90B1* (rho = 0.667; *p* = 0.05) and *ACE* (rho = 0.667, *p* = 0.05). We found significant inverse correlations at 12 months between %change in TNFα and the following proteins: *EPHX2* and *ACE* (for both rho = −0.783, *p* = 0.013). We also found significant inverse correlations between %change in CRP at 12 months and *SHBG* (rho = −0.759, *p* = 0.029), *L1CAM* (rho = −0.904, *p* = 0.002) and *AMBP* (rho = −0.684, *p* = 0.042). Conclusion: Using SWATH-MS, we identified several proteins that are involved in the inflammatory response whose levels change in patients who achieve remission of T2DM after bariatric surgery in tandem with changes in IL6, TNFα and/or CRP. Future studies are needed to clarify the underlying mechanisms in how MBS decreases low-grade inflammation.

## 1. Introduction

Obesity is recognized as the fastest growing problem affecting public health worldwide and has many consequences [1,2,3]. Obesity often has multiple predisposing and precipitating factors and is itself associated with a high mortality rate and with comorbid conditions such as type 2 diabetes mellitus (T2DM), metabolic syndrome, hypertension, dyslipidaemia, several cancers [4], premature cell ageing [5], sleep apnoea and osteoarthritis [6,7]. T2DM is strongly associated with increasing BMI; however, its aetiology remains multifactorial and not completely characterized. Inflammation has been considered an integral part to the development of T2DM in both promoting insulin resistance and beta cell dysfunction [8]. The obese phenotype results in a chronic low-grade inflammatory state characterized by the putative secretion of pro-inflammatory markers by adipocytes [8], including those that may contribute to the inflammatory component in the development of T2DM (IL6, TNFα and IL1β) [9,10,11] by hypertrophic adipocytes.

Metabolic bariatric surgery (MBS) results in metabolic pathway recalibration, which phenotypically can manifest as early remission of T2DM in the days following surgery before any appreciable weight loss has occurred [12]. Additionally, MBS has been shown not only to be an effective therapy for weight loss, but also to improve a variety of metabolic parameters conferring protection from cardiovascular disease (CVD) and other disorders [13,14]. Immediate post-operative weight-independent hormonal changes combined with ensuing weight loss can result in remission of T2DM in up to 80% of patients after one year [14]. MBS results in major metabolic changes and there remain many questions as to the metabolic and inflammatory pathways that are changed following MBS, with significant consequences for health outcomes and how biological phenotype pre-MBS may relate to outcomes post-MBS.

In relation to this, the study of proteins and their function helps investigators to decipher the cellular mechanisms that associate with change in phenotype in relation to specific interventions [15,16]. When major metabolic change occurs, blood protein components have a key role and can be altered significantly. We have recently shown significant changes in the proteome for eight proteins at 6 and 12 months post-BS, and several of these are key components in metabolic and inflammatory pathways [17]. We now set out to identify potential proteins related to inflammatory markers (IL6, TNFα and C-reactive protein—CRP) in plasma samples of people achieving remission of T2DM following MBS.

## 2. Materials and Methods

Longitudinal plasma samples from 10 individuals who achieved remission of T2DM following gastric bypass (*n* = 7) or sleeve gastrectomy (*n* = 3) were analysed. Sequential window acquisition of all theoretical mass spectra (SWATH-MS) proteomics [18] was performed on plasma samples taken at 4 months before (baseline) and 6 and 12 months after MBS, and concurrent analyses of a panel of inflammatory/metabolic parameters were carried out.

All patients were of White ethnicity. Remission was defined as reduction of glycosylated haemoglobin (HbA1c) below 42 mmol/mol with cessation of all antidiabetic therapy at 12 months [19]. SWATH-MS proteomics was carried out on 29 plasma samples with the following sample subgroups: baseline samples, pre-bariatric surgery samples (taken at 4 months prior to MBS (*n* = 10); post-bariatric surgery samples, 6 months follow up (*n* = 10); and post-bariatric surgery samples, 12 months follow up (*n* = 9).

Details of the SWATH-MS analysis are described in our previous publication [17].

Percentage changes were calculated for each of these parameters at 6 and 12 months compared to baseline.

### 2.1. Identification of Proteins

SWATH-MS characterised a total of 467 proteins that changed after bariatric surgery using analysis performed in R and the bioconductor (release 3.5) packages SWATH2Stats (Bioconductor, https://www.bioconductor.org) and MSstats (Bioconductor, https://www.bioconductor.org). We identified 38 proteins with significant fold changes between baseline and 6 months, and 25 proteins with significant fold changes between baseline and 12 months [17]. The detailed methods for SWATH-MS analysis were described previously [17]. In summary, plasma samples (10 µL) were immunodepleted and concentrated afterwards using protein-depletion spin columns and Amicon filters, respectively. Each sample was reduced, alkylated and digested prior to lyophilisation. Samples were then analysed by SWATH-MS with a microflow LC-MS system comprising an Eksigent nanoLC 400 autosampler and an Eksigent nanoLC 425 pump coupled to a SCIEX 6600 Triple-TOF (SCIEX, Framingham, MA, USA) mass spectrometer with a DuoSpray ion source.

We constructed correlation matrices between percentage changes in inflammatory markers (CRP, IL6 and TNFα) and fold changes in significant proteins identified by SWATH-MS at each time point of 6 and 12 months, as described above.

### 2.2. Determination of Glycosylated Haemoglobin (HbA1c), Glucose, Insulin and Inflammatory Markers

HbA1c was measured on an Hb9210 Premier autoanalyser (boranate affinity and high-performance liquid chromatography (Menarini Diagnostics, Wokingham, UK)). Glucose and insulin were measured using Abcam ELISA kits (Abcam, Cambridge, UK), and the HOMA2 calculator (https://www.dtu.ox.ac.uk/homacalculator/) was used to calculate the homeostatic model assessment of insulin resistance (HOMA-IR) and beta cell function (HOMA-B) [20].

The inflammatory markers (interleukin6 (IL6), tumour necrosis factor-alpha (TNFα) and C-reactive protein (CRP) were measured using ELISA kits (Human IL-6, TNFα or CRP Quantikine HS ELISA Kit, R&D Systems, Minneapolis, MI, USA) according to the manufacturer’s instructions.

### 2.3. Ethics

Informed consent was obtained from each participant before recruitment. This research adhered to the tenets of the Declaration of Helsinki. All participants provided written informed consent. The study was approved by the Greater Manchester Research Ethics Committee (REC No:11/NW/0731, IRAS ID: 85208).

### 2.4. Statistical Analysis

Statistical analysis was carried out on SPSS for Mac (Version 23.0, IBM Corporation, New York, NY, USA). Normality was determined by using the Shapiro–Wilk test and by visualising the histogram and normal Q–Q plot. To assess within- and between-group differences, we used one-way analysis of variance for parametric variables and Friedman’s test for non-parametric variables. A significant *p* value was considered to be <0.05 (post hoc Tukey).

We tested for correlations of those plasma proteins with significant fold change difference at each time point (6 and 12 months after MBS) and percentage (%) change in inflammatory markers (IL6, TNFα and CRP). Correlational analysis was done using Spearman’s rank correlation coefficient as all variables were non-parametrically distributed.

## 3. Results

Anthropometric and clinical results are displayed in Table 1. BMI (*p* < 0.001) and HbA1c (*p* < 0.001) declined significantly at both 6 and 12 months. IL6 decreased significantly only at 12 months (t = 2.585, *p* = 0.029), and there were no differences observed between baseline vs. 6 months (*p* = 0.160). CRP, TNF-α, HOMA-IR and HOMA-B declined numerically but did not show statistical significance at any time points.

The list of proteins that correlated with inflammatory markers in relation to fold change at specific time points are shown on Table 2. In particular, L1 cell adhesion molecule (L1CAM) and sex hormone-binding globulin (SHBG) levels increased while heat shock protein 90 Beta family member 1 (HSP90B1) and heat shock protein 4 (HSPA4) levels fell.

### Changes in Inflammatory Markers vs. the Proteome

We found significant correlations between the %change in IL-6 and fold change in several proteins of relevance to inflammation.

**Figure 1:**
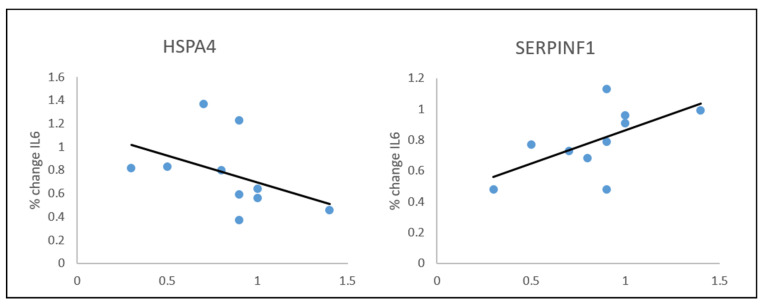
Spearman’s correlations between IL6 % change and plasma proteins at 6 months post-bariatric surgery.

At 6 months: there was a correlation between %change in IL-6 and fold change in *HSPA4* (rho = −0.659; *p* = 0.038) and in *SERPINF1*: rho = 0.714, *p* = 0.020 (Figure 1).

**Figure 2:**
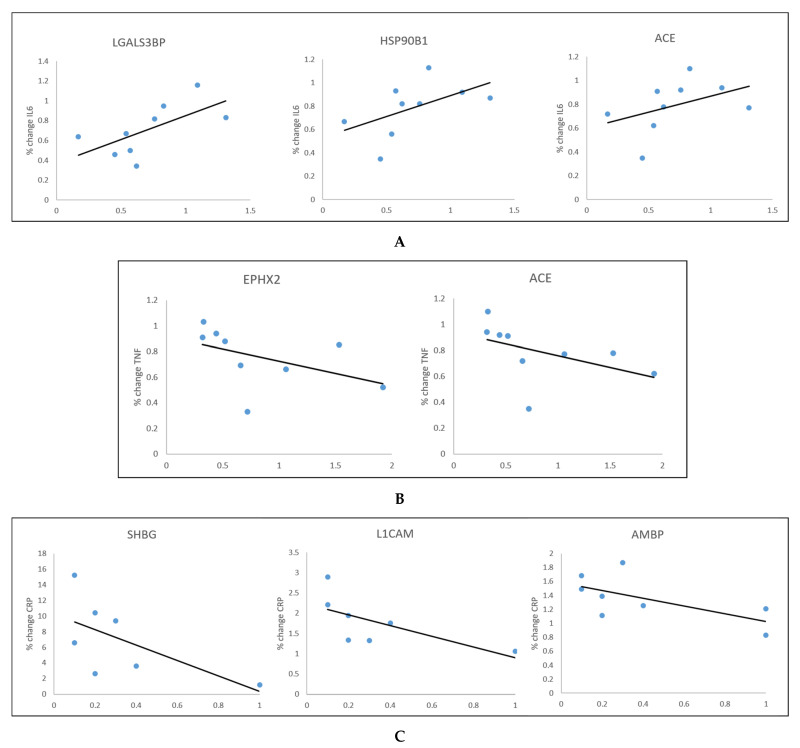
Spearman’s correlations between IL6 (**A**), TNFα (**B**) and CRP (**C**) and plasma proteins at 12 months post-bariatric surgery.

At 12 months: there was a positive correlation between %change in IL-6 and the fold change in the following proteins—*LGALS3BP*: rho = 0.700, *p* = 0.036; *HSP90B1*: rho = 0.667, *p* = 0.05; *ACE*: rho = 0.667, *p* = 0.05 (Figure 2A).

In relation to TNFα, we found significant correlations only at 12 months. These were inverse correlations between %change in TNFα and the following proteins—*EPHX2* and *ACE*—for both rho = −0.783, *p* = 0.013 (Figure 2B).

We also found significant inverse correlations between %change in CRP at 12 months and *SHBG* (rho = −0.759, *p* = 0.029), *L1CAM* (rho = −0.904, *p* = 0.002) and *AMBP* (rho = −0.684, *p* = 0.042) (Figure 2C).

## 4. Discussion

In this study, we identified key proteins that change after bariatric surgery in association with changes in traditional inflammatory markers (IL6, CRP and TNFα) in 10 patients who experienced remission of T2DM. IL6 declined significantly at 12 months compared to baseline. We did not find a statistically significant decline in CRP or TNFα. However, we attribute this to the relatively low number of samples, as previous studies have confirmed that these markers consistently reduce after metabolic bariatric surgery [21], and for this reason, we opted to include these markers in our correlation analysis.

Bariatric surgery brings multiple benefits for severe obesity. The reduction of weigh relates to reduction of comorbidities, such as cardiovascular disease, T2DM, dyslipidaemia, hypertension and low-grade inflammation [13,14]. Subjects undergoing MBS show reduction in various inflammatory indicators, such as CRP and IL6, accompanied by improvements in insulin sensitivity and cardiovascular risk [14]; however, these findings are not always consistent, due to the variability of procedures.

The percentage decline at 6 months after surgery in IL6 correlated with the fold change in *HSPA4* and with *SERPINF1. HSPA4* functions as a cytosolic chaperone involved in facilitating protein folding, degradation, complex assembly and translocation. *HSPA4* appears to be induced by states of chronic inflammation [22]. Previous work has shown elevation of *HSPA4* to occur in tandem with length of time since diagnosis in type 2 diabetes mellitus (T2DM) [23]. *HSPA4* has also been associated with inhibition of nitric oxide production correlating with CRP levels in individuals with T2DM [19]. Styger et al. demonstrated reduction in both serum and liver *HSPA4* in a murine model after MBS [24]. Our work demonstrates a significant reduction in *HSPA4*, which correlated with percentage change in IL6 between baseline and 6 months; however, this correlation was not maintained at 12 months. Further work is required to determine the role of this protein in the pathophysiology of T2DM.

*SERPINF1* is a neurotrophic protein among the most abundant glycoproteins secreted by adipocytes [25] and its genetic variants have been previously associated with obesity, insulin resistance, overall adiposity and circulating leptin levels [26]. Previous proteomic work has demonstrated a decline in this protein in association with inflammatory markers [27]. Administration of *SERPIN1F* has been shown to have anti-inflammatory effects in the progression of diabetic nephropathy and amelioration of albuminuria in a murine streptozocin-induced diabetic model [28]. Our results show a decline in *SERPIN1F* in association with a decline in IL6, suggesting that *SERPIN1F* may be marker of inflammatory resolution in our bariatric cohort.

The decrease seen at 12 months post-MBS vs. baseline in IL6 was correlated positively with fold changes in *LGALS3BP*, *HSP90B1* and *ACE*. *LGALS3BP* is a secreted protein that interacts with its ligand to promote cell–cell adhesion that can initiate pathologic and pro-inflammatory signals [29,30]. A number of studies have noted its role in cancer pathogenesis, and its role as cancer biomarker has been suggested [31,32,33]. Melin et al. noticed that higher levels of this protein were associated with elevated total cholesterol and lower HDL, especially in women [34]. Chen et al. also found that elevation of *LGALS3BP* was an independent risk factor for impaired insulin insensitivity [35]. In a prospective cohort study of 2922 individuals, *LGALS3BP* was found to be independently associated with all-cause and cardiovascular mortality [35]. The authors also found significant associations with other markers of metabolic distress and inflammation. Our study demonstrated a significant decline in *LGALS3B* in association with decline in IL6 discovered for the first time in a bariatric model.

*HSP90B1* is an essential immune chaperone to regulate both innate and adaptive immunity and has previously been shown to be increased in a zebrafish model of hepatic steatosis induced by high-fat and high-cholesterol diet [36]. *HSP90B1* gene expression was reduced in the hippocampus of a murine model of type 2 diabetes [37]. This protein also increases the protective heat shock response by suppressing inflammatory signalling pathways in several diseases [38], and it was previously shown that *HSP90B1* can increase the IL6 secretion in treated N9 microglial cell line [39], which is in accordance with our findings.

Another interesting correlation was between reduction in IL6 and fold change in *ACE*. The role and importance of *ACE* inhibitors in cardiovascular disease related to obesity and diabetes is well known [40]. *ACE* inhibitors are effective not only as blood pressure-lowering agents, but also as modulators of metabolic abnormalities. Indeed, experimental evidence indicates that in animal models of insulin resistance, *ACE* inhibitors ameliorate the insulin resistance [40].

Lower circulating *SHBG* levels are associated with insulin resistance [41,42] and it is well documented that *SHBG* levels increase after bariatric surgery [43]. As would be expected, we found an inverse correlation between change in *SHBG* (increase) and change in CRP (decrease) at 12 months post-surgery.

The other correlations we found between inflammatory markers (TNFα and CRP) with plasma proteins are interesting. However, we consider them as exploratory findings, as we did not find statistical differences in those inflammatory markers comparing baseline and 6 or 12 months post-surgery results. However, it is important to highlight that the proteins correlated are involved in process as immunity/pro-inflammatory activity [44] and neurocognitive functions [45]. This raises the necessity of further studies in this area to validate and confirm our findings.

Our study contributes and helps to give new direction to the study of proteins with possible clinical relevance to the reduction seen in chronic inflammation post-bariatric surgery. It has early implications for the development of strategies to reduce low-grade inflammation and cognitive impairments in obesity.

## 5. Conclusions

Using SWATH-MS, we identified several proteins that are involved in the inflammatory response that declines in patients who achieve remission of T2DM after metabolic bariatric surgery in tandem with changes in IL6, TNFα and/or CRP. Despite a limited sample size, we identified key proteins involved in remission from T2DM after MBS. Replication of these results is now needed together with future studies to understand the mechanisms whereby MBS can improve low-grade inflammation.

## Figures and Tables

**Table 1:** Anthropometric and clinical results from (*n* = 10) individuals having undergone metabolic bariatric surgery (MBS) at baseline and 6 and 12 months after MBS.

	Baseline (mean ± SEM)	6 Months Post-MBS (mean ± SEM)	12 Months Post-MBS (mean ± SEM)	*p* Value (Baseline × 6 Months)	*p* Value (Baseline × 12 Months)
BMI	54.9 ± 3.26	41.8 ± 2.20	38.6 ± 1.60	<0.0001 *	<0.0001 *
HbA1c	56.0 ± 4.46	40.0 ± 1.70	36.44 ± 1.01	0.002 *	0.004 *
IL6	6.29 ± 1.19	4.8 ± 0.96	4.57 ± 1.10	0.160	0.029 *
TNFα	5.98 ± 0.70	5.27 ± 1.20	4.59 ± 0.67	0.689	0.245
CRP	9.07 ± 2.45	7.14 ± 1.71	5.11 ± 1.88	0.226	0.180
HOMA-IR	0.56 ± 0.17	0.48 ± 0.19	0.36 ± 0.08	0.741	0.380
HOMA-B	331.86 ± 83.69	413.85 ± 104.60	376.43 ± 72.88	0.443	0.734

Results were calculated by a paired T test and considered significant when* *p* values ≤ 0.05.

**Table 2:** Fold change in relevant proteins that showed correlations with inflammatory markers at 6 or 12 months post-surgery.

Protein (Gene Symbol)	Fold Change ± SEM at 6 Months	Fold Change ± SEM at 12 Months
Heat shock protein 4 (*HSPA4*)	0.7648 ± 0.10156	N/S
Serpin family F member 1 (*SERPINF1*)	0.7906 ± 0.06724	N/S
Galectin 3 binding protein (*LGALS3BP*)	N/S	0.7074 ± 0.08600
Heat shock protein 90 Beta family member 1 (*HSP90B1*)	N/S	0.7858 ± 0.07666
Angiotensin-converting enzyme (*ACE*)	N/S	0.7888 ± 0.07232
Epoxide hydrolase 2 (*EPHX2*)	N/S	0.7587 ± 0.07564
Sex hormone-binding globulin (*SHBG*)	N/S	6.1816 ± 1.83129
L1 cell adhesion molecule (*L1CAM*)	N/S	1.6862 ± 0.22992
Alpha-1-microglobulin/Bikunin precursor (*AMBP*)	N/S	1.3137 ± 0.11014 *

N/S non-significant. * *p* ≤ 0.05.

## Data Availability

Any requests for data extracts will be considered by Adrian Heald.
